# A Case of Lacrimo-Auriculo-Dento-Digital Syndrome with Multiple Congenitally Missing Teeth

**DOI:** 10.1155/2016/8563961

**Published:** 2016-10-10

**Authors:** Lumbini Pathivada, Munagala Karthik Krishna, Mandeep Rallan

**Affiliations:** ^1^Department of Paedodontics and Preventive Dentistry, Teerthanker Mahaveer Dental College and Research Centre, Moradabad, India; ^2^Department of Periodontology, Teerthanker Mahaveer Dental College and Research Centre, Moradabad, India

## Abstract

Lacrimo-auriculo-dento-digital (LADD) syndrome is an extremely rare disorder which may occur sporadically or inheritably as an autosomal dominant condition. It is characterized by defects in the lacrimal apparatus, ear problems, and dental and digital abnormalities. However, specific symptoms vary greatly among the cases with a high degree of overlap with other similar genetic disorders. Here, we describe a 7-year-old boy with LADD syndrome, clinical and radiological findings, dental treatment undertaken, and its differential diagnosis.

## 1. Introduction

Lacrimo-auriculo-dento-digital (LADD) syndrome, also known as Levy-Hollister syndrome, is a rare genetic disorder characterized by anomalies affecting the lacrimal and salivary glands and ducts, ears, dentition, and extremities with a high degree of variation in the signs and symptoms and an overlap of similar findings in other genetic disorders [1–3]. Malformations in the lacrimal apparatus, including hypoplastic or aplastic lacrimal puncta and/or an obstruction of the nasolacrimal duct, excessive tearing (epiphora), inflammation of the tear sac (dacryocystitis), and dryness and inflammation of the cornea and conjunctiva (keratoconjunctivitis), are common findings in this syndrome. Less commonly, underdeveloped or missing lacrimal glands may result in lack of tears (alacrima) and dry eyes (xerophthalmia) [4–6].

Underdevelopment or absence of the salivary glands may result in dry mouth (xerostomia) and vulnerability to severe dental caries. Other oral findings include small teeth (microdontia), enamel hypoplasia, belated eruption, and missing teeth [7–9]. Cup-shaped, low-set ears maybe present associated with mild to severe hearing loss [1, 2]. Individuals with this syndrome may present variable digital features such as aplasia, hypoplasia, duplication, clinodactyly, partial syndactyly, digitalization, and abnormal placement of fingers [9–16].

## 2. Case Report

**Figure 1:**
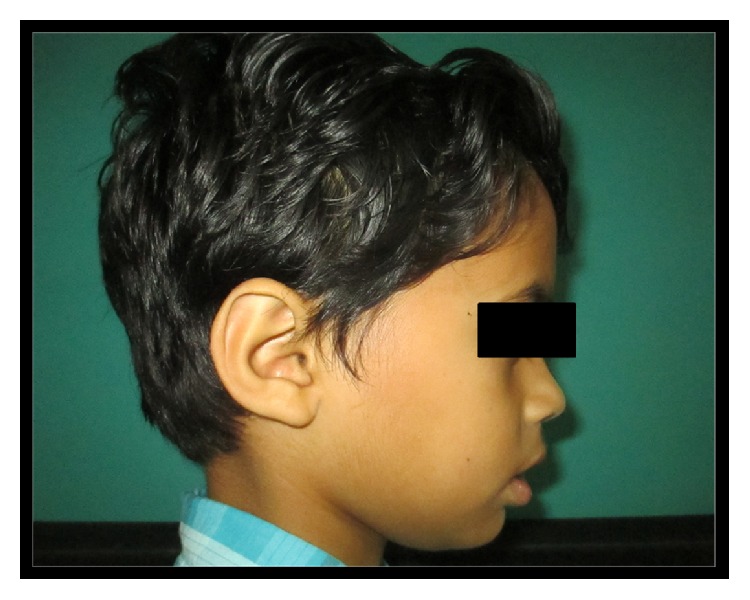
Presence of cup-shaped ear and prominent parietal aspect of head.

A 7-year-old boy reported to the Department of Pedodontics and Preventive Dentistry with a chief complaint of dryness of mouth since birth and decayed teeth. Additional concerns included dryness and itchiness in the eyes. The patient weighed 28 kgs and the measured height was 115.2 cm. He had abundant scalp hair but normal hair density was observed on the body and limbs. His ears appeared to be normally located but were small and cup-shaped. Shape of the head was abnormal in that the parietal aspect appeared prominent although the frontal region was normal (Figure 1). Eyes appeared dry with lack of tearing at the time of examination. Patient reported that his mother and sister experienced similar ocular symptoms although they were not available for examination.

**Figure 2:**
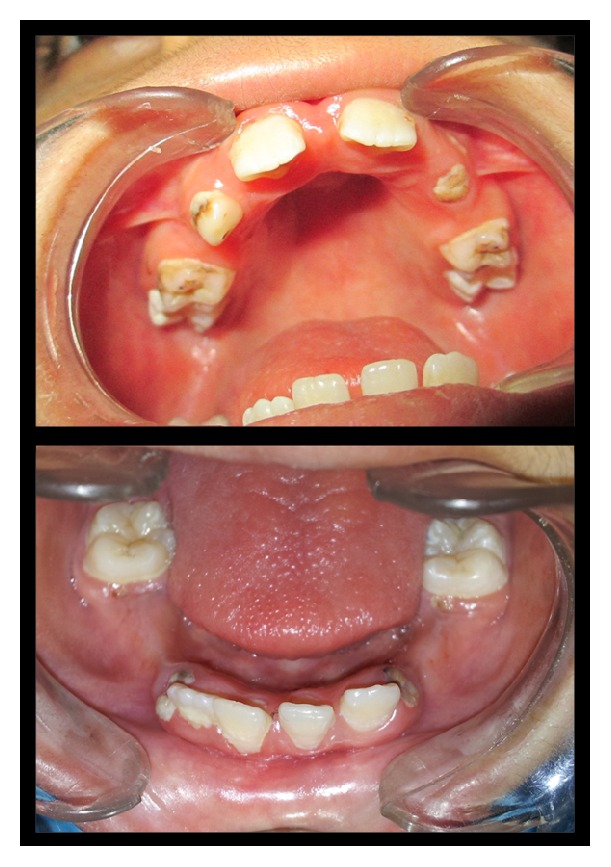
Intraoral view showing multiple carious lesions, reduced saliva, and missing primary dentition.

**Figure 3:**
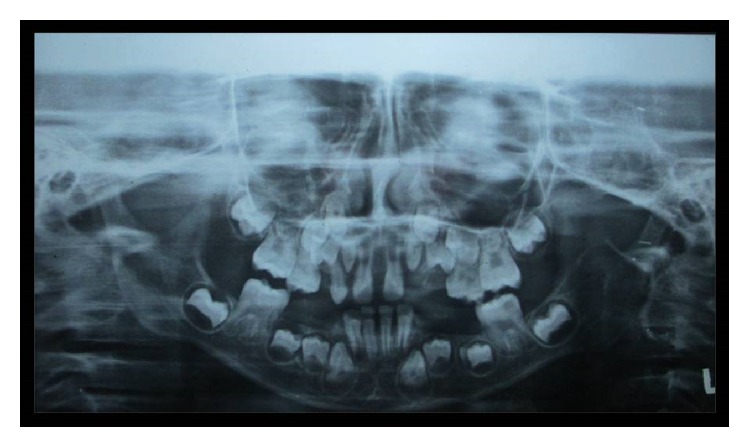
Panoramic radiograph showing lack of primary dentition tooth buds.

**Figure 4:**
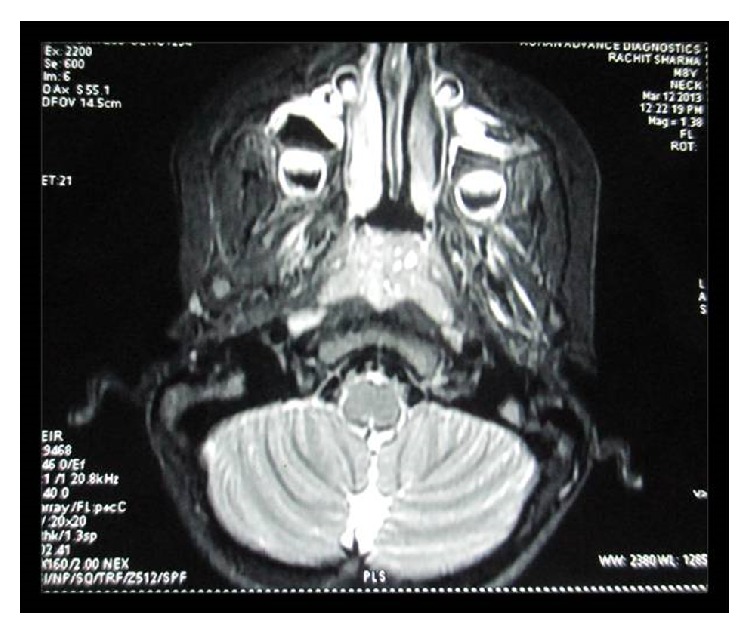
MRI showing aplasia of lacrimal and salivary glands.

Palpation of soft tissues in the submandibular and parotid regions revealed no perceptible enlargement. On intraoral examination, reduced saliva with multiple carious lesions and enamel dysplasia were observed. Several primary teeth were absent as per their chronological age of eruption (Figure 2). A panoramic radiograph confirmed this finding, although tooth buds of unerupted permanent teeth were evident (Figure 3). MRI scan revealed aplasia of bilateral parotid and submandibular salivary glands and hypertrophied minor salivary glands along oropharyngeal wall. Lacrimal apparatus agenesis was also evident (Figure 4).

**Figure 5:**
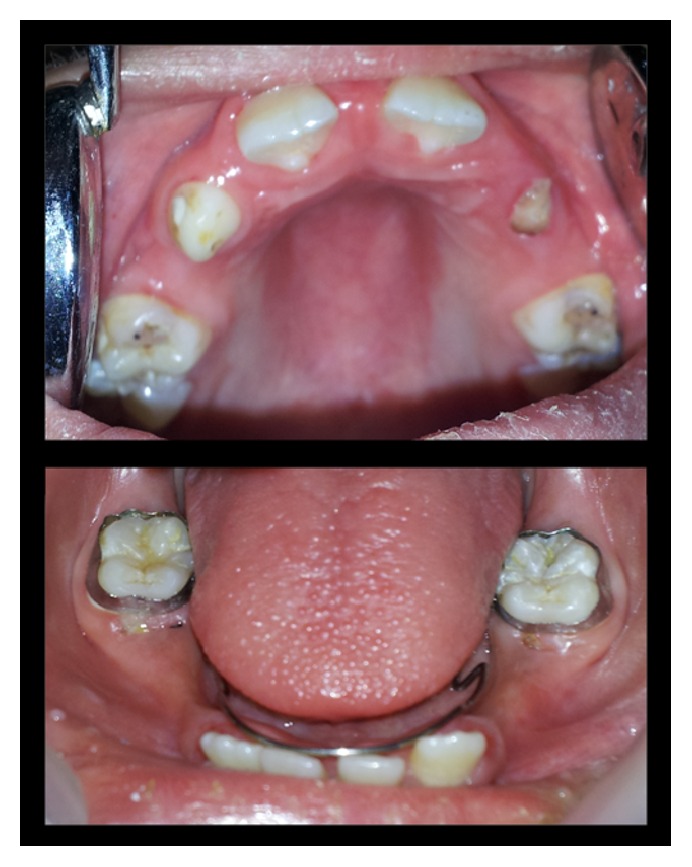
Posttreatment view with restored caries and placement of space maintainer in lower arch.

**Figure 6:**
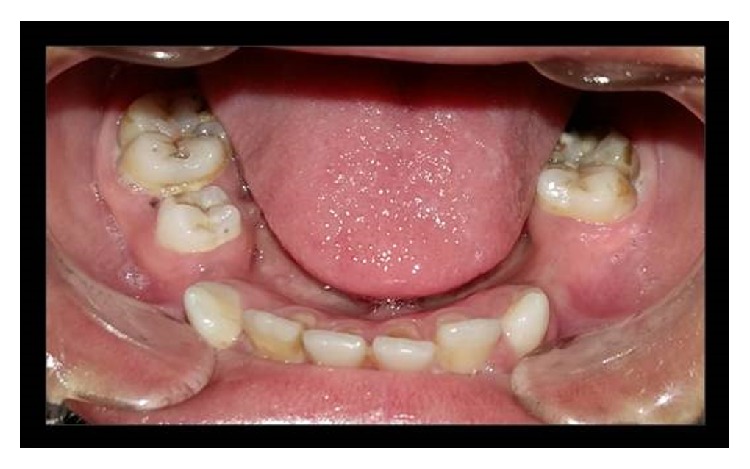
Three-year follow-up view with erupted permanent dentition.

Dental treatment involved scaling, restorations in relation to primary maxillary canines and second molars, and extraction of root stumps of mandibular canines. Band adaptation was done in relation to permanent molars and alginate impressions were taken for the purpose of fabricating space maintainers. Patient was instructed in oral hygiene measures, prescribed fluoride mouthrinse and salivary substitute, and recalled. During the second visit, a lingual arch space maintainer was given in the lower arch, and topical fluoride varnish and resin-based pit and fissure sealants were applied (Figure 5). Patient refused an upper arch space maintainer due to apparent discomfort and was placed on a regular recall protocol. Evaluation after 3 years showed eruption of lower permanent canines and right second premolar (Figure 6).

## 3. Discussion

In this case report, we have described a 7-year-old boy with reduced lacrimal secretions, cup-shaped ears, and dental anomalies, characteristic of LADD syndrome. However, the child lacked significant digital malformations. Reduced tears and saliva production were probably as a consequence of aplasia of lacrimal and salivary glands, respectively, as confirmed by imaging methods. The proband demonstrated bilateral cup-shaped ears that were positioned normally and no concomitant hearing loss.

A significant finding in the present case was the absence of several primary teeth. Maintenance of arch space in such cases presents a clinical challenge due to increased caries risk and discomfort in wearing appliances because of diminished salivary flow. However, in the present case, prescription of salivary substitute may have played a role in enabling the patient to wear the appliance comfortably due to its lubricating properties which proved to be beneficial in that the patient could wear the appliance for a period sufficient to prevent pathological migration and provided space for eruption of permanent teeth.

LADD syndrome is an autosomal dominant disorder caused due to mutations in one of at least three genes, the fibroblast growth factor receptor 2 (FGFR2), fibroblast growth factor receptor 3 (FGFR3), and fibroblast growth factor 10 (FGF10) [11–13]. Several other conditions have common, overlapping clinical features and similar genetic etiology but are yet distinct from LADD syndrome. Aplasia of the lacrimal and salivary glands (ALSG) presents with symptoms including xerophthalmia, xerostomia, scarring of the conjunctiva, dental erosion, periodontal disease, and increased risk of dental caries. ALSG is inherited as an autosomal dominant disorder caused due to mutation in the FGF10 gene [14]. Ectrodactyly-ectodermal dysplasia-cleft lip/palate (EEC syndrome) is another autosomally dominant syndrome and characterized by digital malformations, cleft palate, and cleft lip. EEC individuals may present with features that overlap with the LADD phenotype, including abnormalities of lacrimal ducts, chronic conjunctivitis, hypodontia, and/or microdontia [15]. Labyrinthine aplasia, microtia, and microdontia (LAMM) syndrome presents with Michel aplasia (complete bony and membranous aplasia of the inner ear) in association with microdontia and microtia. LAMM is characterized by an autosomal recessive pattern of inheritance involving mutations in the* FGF3 *gene [16].

LADD syndrome is an extremely rare condition with characteristic oral, lacrimal, and auditory clinical findings. It requires a thorough evaluation to rule out the above-mentioned similar conditions and determine presence of associated systemic manifestations. Multiple missing primary dentition is an unusual association that requires comprehensive dental therapy.
